# Patient perceptions of facilitators and barriers to reducing hazardous alcohol use among people living with HIV in East Africa

**DOI:** 10.1186/s13011-023-00520-7

**Published:** 2023-02-03

**Authors:** Jayne Lewis-Kulzer, Margaret Mburu, Sarah Obatsa, Julius Cheruiyot, Lorna Kiprono, Steve Brown, Cosmas Apaka, Hillary Koros, Winnie Muyindike, Edith Kamaru Kwobah, Lameck Diero, Maurice Aluda, Kara Wools-Kaloustian, Suzanne Goodrich

**Affiliations:** 1 Department of Obstetrics, Gynecology and Reproductive Sciences, University of California San Francisco, 550 16TH Street, 3rd Floor, San Francisco, CA USA; 2 Centre for Microbiology Research, Kenya Medical Research Institute, P.O. Box 1578-40100, Kisumu, Kenya; 3 Academic Model Providing Access to Health Care (AMPATH), P.O. Box 4606-30100, Eldoret, Kenya; 4 Department of Biostatistics and Health Data Science, School of Medicine, Indiana University, 410 W. 10th Street, HITS 3000, Indianapolis, IN USA; 5 The Immune Suppression Syndrome Clinic, Mbarara University of Science and Technology, P.O. Box 40, Mbarara, Uganda; 6 Department of Mental Health, Moi Teaching and Referral Hospital, P.O. Box 3-30100, Eldoret, Kenya; 7 Department of Medicine, Moi University School of Medicine, P.O. Box 4606-30100, Eldoret, Kenya; 8 Division of Infectious Diseases, Department of Medicine, School of Medicine, Indiana University, 545 Barnhill Drive, Indianapolis, IN USA

**Keywords:** HIV, Antiretroviral therapy, Alcohol, Adherence, East Africa

## Abstract

**Background:**

Hazardous alcohol use among people living with HIV is associated with poor outcomes and increased morbidity and mortality. Understanding the hazardous drinking experiences of people living with HIV is needed to reduce their alcohol use.

**Methods:**

We conducted 60 interviews among people living with HIV in East Africa with hazardous drinking histories. Interviews and Alcohol Use Disorder Identification Test (AUDIT) scores were conducted 41 – 60 months after their baseline assessment of alcohol use to identify facilitators and barriers to reduced alcohol use over time.

**Results:**

People living with HIV who stopped or reduced hazardous drinking were primarily motivated by their HIV condition and desire for longevity. Facilitators of reduced drinking included health care workers’ recommendations to reduce drinking (despite little counseling and no referrals) and social support. In those continuing to drink at hazardous levels, barriers to reduced drinking were stress, social environment, alcohol accessibility and alcohol dependency.

**Conclusions:**

Interventions that capacity-build professional and lay health care workers with the skills and resources to decrease problematic alcohol use, along with alcohol cessation in peer support structures, should be explored.

## Background

Hazardous alcohol use and retention in HIV treatment are overlapping public health concerns in sub-Saharan Africa (SSA). Alcohol is the most common form of substance hazardously used in Africa [1–5]. Hazardous alcohol use is linked to a range of medical conditions including HIV, non-communicable diseases, injury and mental health disorders and injury [4–10]. Africa carries the largest global burden of disease and injury attributed to alcohol with 5.1% of all deaths and 4.1% of all disability-adjusted life years (DALYs) lost as well as Alcohol Use Disorder (AUD) affecting 3.7% of the population [4]. Although only about one-third of the adult population drinks alcohol in Africa, the amount consumed among drinkers is high with 50.2% of drinkers being heavy episodic drinkers as compared to 39.5% globally [4]. It is estimated that about 30% of alcohol use is unrecorded in the region with homebrew consumption, indicating that alcohol consumption is likely higher than reported in many sources [2, 11–14]. With hazardous alcohol use on the rise in SSA, where two-thirds of the world’s people living with HIV reside, there is cause for concern [11, 15].

Although widespread antiretroviral therapy (ART) has resulted in sharp declines in new HIV infections and deaths in SSA, engagement in HIV care remains a pressing challenge, and hazardous alcohol use is known to exacerbate non-adherence to ART and non-retention to clinical care [15–20]. People Living with HIV (PLWH) require consistent adherence and retention to lifelong ART to sustain viral suppression for optimal clinical outcomes and to prevent onward transmission [19, 21–23]. However, estimates in low- and middle-income countries show that retention among PLWH on ART drops to 83%, 74%, and 68–69% by 12, 24, and 36 months of follow-up, respectively [24, 25]. Based on the Alcohol Use Disorders Identification Test (AUDIT) scores, a study conducted in Kenya and Uganda found that 28% of PLWH had hazardous drinking behaviors and that drinkers had higher attrition from care rates [18]. Moreover, PLWH have higher prevalence of AUD than the general population, 25% in developing countries, and are more likely to be binge drinkers [26–28]. Hazardous alcohol use among PLWH is associated with poor treatment outcomes, including higher rates of viral non-suppression, morbidity and mortality [20, 29–32]. Alcohol compromises the immune system, contributing to disease progression and leads to negative effects on ART, including drug interactions, toxicity and resistance to ART [9, 33–36].

There has been little investigation into the help seeking behaviors of those with hazardous alcohol use, though there are studies from South Africa that have examined factors associated with alcohol use and its impact on ART adherence. These studies have shown that problem drinking is associated with spending a higher percentage of income on alcohol, being less goal-driven and leisure boredom [37–40]. Non-adherence to ART has been linked to misconceptions about concurrent alcohol use and ART, as well as stigmatization of individuals who drink alcohol [41, 42]. A deeper understanding of PLWH experiences with hazardous drinking in a similar economic but wider geographic context is needed to help identify problematic drinking factors among PLWH on ART and avenues for interventions. This qualitative study explored patient perceptions of facilitators, barriers and recommendations for curbing hazardous alcohol use in East Africa.

## Methods

### Study design

We conducted a qualitative study within the *Alcohol Use Assessment Cohort* (AUAC) (U01AI069911), a longitudinal study examining alcohol use among adult patients in HIV care. This study involved a baseline assessment of alcohol use (AUAC Phase 1) among a cohort of adult PLWH at their time of enrollment into HIV care. Members of this cohort were re-enrolled in the study 41- 60 months after enrollment into HIV care and re-assessed for their clinical outcome and alcohol use (AUAC Phase II) [18].

### Setting

This study took place within the East Africa International epidemiology Databases to Evaluate AIDS (EA-IeDEA) consortium. EA-IeDEA is one of seven regional consortia supported by the National Institutes of Health to consolidate, curate and analyze HIV care and treatment data to evaluate the outcomes of people living with HIV/AIDS [43]. Five EA-IeDEA affiliated clinics participated in this study: Family AIDS Care and Education Services (FACES) supported clinics at Lumumba sub-County Hospital in Kisumu County, Kenya [44] and Suba sub-County Hospital in Homa Bay, Kenya; two Academic Model Providing Access to Healthcare (AMPATH) clinics based at Moi Teaching and Referral Hospital in Eldoret, Kenya; and the Mbarara Immune Suppression Syndrome (ISS) clinic in Mbarara, Uganda. The Eldoret and Kisumu locations are largely urban, while the Homa Bay and Mbarara locations are semi-urban. Each clinic provides comprehensive HIV services in accordance with the national guidelines of their country.

### Study population

Participants were eligible for enrollment in this qualitative study if they were adults (18 years of age and above) living with HIV, and if they were previously enrolled in Phase I of the AUAC and identified with hazardous alcohol consumption (AUDIT score ≥ 8) between January 2013 and June 2014 in the participating clinics in Eldoret, Kisumu, Homa Bay and Mbarara.

### Sampling

Systematic sampling was used to recruit eligible participants from the Phase I AUAC study for this Phase II of the AUAC study. To obtain theoretical saturation on key domains, including self-perception of alcohol use, help-seeking behaviors and barriers to address hazardous drinking, about 30% were systematically sampled (every K^th^ participant sequentially) from among the hazardous drinkers in AUAC (AUDIT score ≥ 8) at baseline [45].

### Data collection

Phase II data was collected July 2017 – July 2018, during the 41 – 60 month AUAC follow-up window, about four years on average after baseline. Trained and experienced research assistants at each site approached potential participants for study recruitment during their routine HIV clinic return visits or traced them in the community if they were lost to follow-up. After receiving informed consent, research assistants administered a one-hour semi-structured interview guide. The interview guide developed by the EA-IeDEA qualitative core team was designed to garner insights on self-perception of alcohol use, including consequences of drinking and barriers and motivations to reduce problematic drinking. Help-seeking behaviors and advice received to address alcohol use was also included in the guide. The English guide was translated into the local languages Kiswahili, Dholuo and Rukiga/ Runyankole by professional translators and back translated by study staff fluent in the local language. The most widely spoken languages specific to each location were utilized: Kiswahili and English in Eldoret, Dholuo, Kiswahili and English in Kisumu, and English and Rukiga/Runvankole in Mbarara. The interviews were conducted in a private setting within each health facility in the participant’s preferred language and audio-recorded with the participant’s permission. The AUDIT follow-up was administered on the same day, just after the interview in Phase II of the AUAC study.

Descriptive data were abstracted from electronic medical records at enrollment, including age, gender, marital status and World Health Organization (WHO) clinical stage – a 4-stage clinical assessment system of HIV-related disease severity (WHO Stage 1: asymptomatic, Stage 2: mild symptoms, Stage 3: advanced symptoms, and Stage 4: severe symptoms) [46]. AUDIT scores at baseline and follow-up were retrieved from a study-specific REDCap database.

### Data analysis

The primary qualitative aims are presented in this paper. The qualitative interview translations and transcriptions were conducted by research assistants who were fluent in English and native speakers of the local languages (Dholuo, Kiswahili, Rukiga/Runyankole) and trained in qualitative research methods. The process involved directly translating from the local language into English while transcribing the audio recording. Upon completion of each transcription, a second research assistant validated the accuracy of the transcription by listening to the audio recording while reading the transcript. Any changes were discussed and made jointly. The transcriptions were uploaded to MAXDQA for coding. Transcripts were coded both deductively and inductively using a theory-informed coding framework based on the interview guide domains. Domains centered on self-perception of alcohol use, health-seeking behavior facilitators, barriers, and enablers, and recommendations to reduce problematic drinking.

Baseline descriptive statistics were generated in Stata Statistical Software (*StataCorp. 2017. Stata Statistical Software: Release 15. College Station, TX: StataCorp LLC*) to characterize the cohort of participants in this qualitative study, including their demographics and their AUDIT scores at baseline and follow-up. The AUDIT measure has a minimum score of 0 (non-drinkers) and a maximum score of 40. According to WHO guidelines, a score of 1 to 7 suggests low-risk alcohol consumption (nonhazardous), a score of 8 to 14 suggests hazardous or harmful alcohol consumption, and a score of 15 or higher indicates the likelihood of alcohol dependence (moderate-severe alcohol use disorder) [47].

In this study, AUDIT scores at baseline and follow-up were compared to see if participant alcohol use had changed from their hazardous level at baseline to help us better understand the barriers and motivators to curbing alcohol use when analyzing the qualitative data. Participants were categorized into three groups: no longer a hazardous drinker (AUDIT ≤ 8 at follow-up), drinking less but still at a hazardous level (AUDIT score decreased to a score of 8 to14 at follow-up from a score of 15 or higher at baseline), and those that had maintained or increased their score category between baseline and follow up: staying in the hazardous range (8 to 14) or alcohol dependence range (≥ 15) or increased to the alcohol dependence range (increased to ≥ 15 from 8 to 14 at baseline).

A six-person qualitative team, including data collectors, research coordinators and a co-investigator, participated in the analysis and interpretation of the data. Codes were queried and resulting excerpt segments were read and summarized independently by each team member. The qualitative team met several times to review excerpts and summaries to detect patterns, resolve discrepancies and identify key themes using the grounded theory approach [48]. The qualitative transcripts were then matched to the AUDIT drinking level and the analytical process was repeated to distinguish facilitator and barrier themes and nuances based on alcohol use consumption at follow-up.

## Ethical approval

This study was approved by the Indiana University IRB (#1212010134) and the regulatory bodies affiliated with each participating site: AMPATH: Moi University College of Health Sciences and Moi Teaching and Referral Hospital’s Institutional Research and Ethics Committee (IREC) in Eldoret, Kenya (#0001922); the ISS University of Science & Technology Institutional Review Committee in Mbarara, Uganda (#09/12–12); and the Kenya Medical Research Institute (KEMRI) Scientific Ethics Review Unit (SERU) in Nairobi, Kenya (#3708). Written informed consent was obtained from participants at the time of study enrollment.

## Results

### Demographics and alcohol use characteristics

Phase I of the AUAC study enrolled 765 participants with 204 hazardous alcohol users. Sixty-four participants were systematically sampled to participate in this study, of which four were found to have a non-hazardous AUDIT score < 8 at baseline and were dropped during data analysis. Data for 60 PLWH (36.7% female) were analyzed for this study (Table 1). Twenty-nine (48.3%) were from Kisumu, 28 (46.7%) were from Eldoret and three (5.0%) were from Mbarara. Participants ranged in age from 23 to 73 years with a median age of 39 years.

**Table 1:** Participant demographic characteristics by sex

**Variable**	**Female (*n* = 22);** ***n (%), median/mean (min max)***	**Male (*n* = 38);** ***n (%), median/mean (min max)***	***N* = 60;** ***n (%), median/mean (min max)***
**Facility**			
Eldoret	12 (54.5)	16 (42.1)	28 (46.7)
Kisumu	10 (45.5)	19 (50.0)	29 (48.3)
Mbarara	0	3 (7.9)	3 (5.0)
**Median age at follow-up (years)**	33.9 (23.2, 65.7)	42.2 (29.5, 72.9)	39.8 (23.2, 72.9)
**Marital status**			
Never Married and Not Living w/Partner	3 (15.0)	0 (0)	3 (5.3)
Legally Married	8 (40.0)	34 (91.9)	42 (73.7)
Separated	6 (30.0)	1 (2.7)	7 (12.3)
Divorced	1 (5.0)	1 (2.7)	2 (3.5)
Widowed	2 (10.0)	1 (2.7)	3 (5.3)
* Missing*	2 (9.1)	1 (2.6)	3 (5)
**HIV Disclosure**			
No	10 (47.6)	12 (36.4)	22 (40.7)
Yes	11 (52.4)	21 (63.6)	32 (59.3)
* Missing*	1 (4.5)	5 (13.2)	6 (10)
**WHO Stage**			
1	12 (60.0)	22 (61.1)	34 (60.7)
2	6 (30.0)	6 (16.7)	12 (21.4)
3	2 (10.0)	5 (13.9)	7 (12.5)
4	0 (0)	3 (8.3)	3 (5.4)
*** Missing***	2 (9.1)	2 (5.3)	4 (6.7)

The baseline AUDIT scores of those interviewed ranged from 8 to 36, with a mean of 19 (Table 2). Scores from the follow-up AUDIT ranged from 0 to 37, with a mean of 13. At follow-up, 18 (30.0%) participants had reduced their AUDIT scores to ≤ 8, suggestive of non-hazardous drinking, including 11 (61.1%) who had stopped drinking completely. This group was predominantly female. Forty-two (70.0%) participants continued to drink at a hazardous level (AUDIT scores ≥ 8), including 8 (19.0%) who had decreased their hazardous drinking level and 34 (81.0%) who maintained or increased their hazardous drinking level.

**Table 2:** Alcohol AUDIT Scores at baseline and follow-up by sex

**Variable**	**Female (*n* = 22);** ***n (%), median/mean*** **(min max)**	**Male (*n* = 38);** *n (%), median/mean* ***(min max)***	***N* = 60;** ***n (%), median/mean*** ***(min max)***
**Initial alcohol AUDIT** *(mean)*	17.4 (8, 33)	20.2 (8, 36)	19.2 (8, 36)
**Follow-up alcohol AUDIT** *(mean)*	10.2 (0, 37)	15.1 (0, 35)	13.3 (0, 37)
**Alcohol consumption at follow-up**			
** Hazardous**	**11/42 (26.2)**	**31/42 (73.8)**	**42 (70.0)**
Decreased drinking level	2/11(18.2)	6/31 (19.4)	8 (19.0)
Same drinking level	9/11 (81.8)	23/31 (74.2)	32 (76.2)
Increased drinking level	0/11 (0)	2/31 (6.4)	2 (4.8%)
**Non-hazardous**	**11/18 (61.1)**	**7/18 (38.9)**	**18 (30.0)**
Stopped completely	8/11 (72.7)	3/7 (42.9)	11/18 (61.1)
Reduced	3/11 (27.3)	4/7 (57.1)	7/18 (38.9)

### Qualitative findings

#### Patient perception of the negative consequences of alcohol use

The major themes identified as negative consequences of heavy alcohol use were the disruptive effects to ART adherence and health, work interruptions, financial hardship and family troubles. Participants frequently mentioned that drinking interfered with their ART adherence, causing them to delay taking their treatment or forgetting it all together.

*“Sometimes you may go drinking alcohol then forget the time for taking [ART] medication.”* (40 years old, male, Kisumu, AUDIT scores 27 to 25)

Poor self-care (e.g. eating poorly, oversleeping, feeling unwell) was commonly attributed to alcohol use. ART adherence interruptions and poor self-care were generally among those who continued to drink at the same or increased hazardous level.

*“[weeping] I don’t even do any work, they prepare alcohol at my home and when people start gathering to drink I always join them and drink also. At around ten in the morning I go back to sleep and wake up by two in the afternoon and continue drinking again. When night falls, I go back to sleep and I don’t even eat anything, and I do that every other day”* (35 years old, female, Eldoret, AUDIT scores: 33 to 27)

Participants also indicated that their alcohol use commonly led to interruptions in their work life, such as missing or being late for work.

“…*when you drink late into the night you find yourself unable to wake up early so I missed work most of the time.”* (54 years old, male, Eldoret, AUDIT scores: 17 to 14)

The toll alcohol takes on economic and family well-being also emerged as a common negative consequence, with income loss or the misuse of income on alcohol rather than on household essentials, and the resulting financial hardship.

### Patient perception of facilitators to reduce alcohol use

The primary facilitator identified to reduce alcohol consumption was participants’ desire to adhere well to ART for their health and longevity. Among those who had reduced to non-hazardous drinking levels or stopped drinking completely, receiving a diagnosis of HIV infection prompted them to make a conscious decision to change their drinking behavior. Patients recognized that their alcohol consumption interfered with their ability to adhere to treatment, realized drinking was taking a toll on their health, and that they wanted improve their health and longevity. HIV infection provided a strong life or death reason to curtail alcohol use. Consequently, these participants reported high levels of adherence to their ART and subsequent satisfaction with the positive changes in their life after drinking less.

*“When I started taking these drugs-ARVs, that’s when I quit taking alcohol.”*

(65 years old, female, Eldoret, AUDIT scores: 14 to 0)

*“I was told not [to] use alcohol and at the same time am using ARVs because the medicines won’t be effective. So I made my decision of stopping to take alcohol.”* (25 years old, Male, Kisumu, AUDIT scores: 33 to 0)

“*I quit it myself to prolong my life span.”* (58 years old, female, Eldoret, AUDIT scores: 10 to 0)

The desire to adhere well prevailed among those who were drinking less but were still at a hazardous level; their HIV status prompted them to cut down on alcohol consumption. Although many indicated wanting to stop completely, they found it extremely difficult.

*“I have come from far, from those days of like 10 bottles coming down to 3 or 4.”* (37 years old, male, Kisumu, AUDIT scores: 18 to 8)

A few perceived that they could function well at their current reduced level and used strategies such as putting limits on their drinking.

“*Before I used to take too much, but currently I feel I am okay because even if I drink, I still do my work as required and when I wake up in the morning I feel just fine*. *I can only take like three if I am with friends but if I am alone, I can stay without alcohol for even a month.*” (43 years old, male, Kisumu, AUDIT scores: 22 to 11)

Participants’ decisions to change their drinking behavior were also facilitated by the recommendation of health care workers. Participants in all groups (non-hazardous, still hazardous but drinking less, and those who maintained or increased hazardous drinking) learned from health care workers that alcohol could interfere with ART adherence and functionality. The recommendation at the clinic generally came from a clinician or nurse in the lead-up to ART initiation. However, in nearly all cases, there were no further follow-up discussions or referrals for support.

*“I was counseled here at the clinic, but I was not sent anywhere else for help”. They [health care workers at the clinic] told me to stop drinking alcohol since I was going to be initiated on ART and that alcohol could make me forget to take medicine on time or after defaulting on ART the drugs may stop working.”* (43 years old, male, Kisumu, AUDIT scores: 22 to 11)

*“I made a decision [to stop] based on what the clinician told me and being a single mother, I wanted to take good care of my children so that they can have a better life.”* (49 years old, female, Eldoret, AUDIT scores: 12 to 0)

Among those who had reduced to non-hazardous levels of drinking or were drinking less but at hazardous levels, social support emerged as an important facilitator to reducing alcohol consumption. Social support from family, friends and the community motivated participants to curtail drinking to improve family life, their future and their children’s future. Many participants had family members who provided encouragement and support to reduce their drinking. Several were urged by spouses and children to stop for their families and to be better role models.

“*She [my daughter] tells me not to drink so as to be able to take good care of my family since I am the only breadwinner in the house.*” (56 years old, female, Eldoret, AUDIT scores: 15 to 5)

“*It was my wife. She used to tell me to quit or even reduce the amount of alcohol I was taking because I used to come to the house drunk and the kids were there as I’d stagger in*.” (50 years old, Male, Eldoret, AUDIT scores: 27 to 7)

Social support from friends and the community (e.g. church) was also influential with their advice and ongoing support. Social supporters wanted the participants to address their problem to become better people and accomplish more in their lives. One man spoke about the value of social support and the encouragement his support network provided to him during his journey to reduce his alcohol use. Social support was more commonly mentioned among those who had reduced their hazardous drinking.

*“The church is always present in our neighborhood; they have been helpful for me so many times. They always visited me once or twice a month to encourage and give me hope.”* (54 years old, male, Eldoret, AUDIT scores: 17 to 14)

The desire for improved livelihood and economic security were also identified as facilitators to reducing alcohol consumption among non-hazardous drinkers and those drinking less but at a hazardous level. Participants described wanting to prioritize their finances better. They indicated that their income was “hard-earned” and worried it was being spent unwisely on alcohol, constrained finances at home, and lead to quarrels and hardship. They also did not want to lose income by missing or losing work.

*“I feel that it is not okay because sometimes I spend all the money that I have and find myself without any money when I wake up the next day, then end up having problems with my wife.”* (40 years old, male, Kisumu, AUDIT scores: 27 to 25)

### Patient perception of barriers and enablers that impede ability to reduce alcohol use

Among those drinking at the same hazardous or increased hazardous level, alcohol dependency emerged as the primary barrier to reducing alcohol use, despite participants’ desire and attempts to curtail drinking and adhere to ART. They prioritized their treatment for HIV but were unable to stop drinking and revealed missing ART doses due to their drinking habit.

“*I do find that it is a problem [alcohol use], bearing in mind that I am HIV positive and also a fisherman. I have tried to reduce and stop but I can’t, I just can’t (looks down).”* (33 years old, male, Suba, AUDIT scores: 15 to 28)

*“I am trying to stop drinking but whenever I see someone drunk, I just feel like drinking also.”* (32 years old, female, Kisumu, AUDIT scores: 20 to 12)

Stress was identified as a common barrier to reducing alcohol use. Daily stress fueled by work dynamics (e.g. pressure, long hours), family life (e.g. divorce, loss, money concerns), livelihood challenges (e.g. job loss), and coping with their diagnosis of HIV led to self-medicating behaviors. Most participants recognized that drinking was a short-term coping mechanism and realized that their stress re-surfaced once the alcohol had worn off.

*“Okay, I go drinking when something is bothering me. When I have 2 or 3 beers I get sleep but when I wake up in the morning the stress is still there.”* (36 years old, female, Eldoret, AUDIT scores: 11 to 10)

The social environment emerged as a common barrier and enabler impeding the ability to reduce alcohol consumption. The social expectations to drink at parties, nightclubs, barbeques and weddings were commonly mentioned; with drinking being expected as part of socializing, celebrating and relaxing with friends and family. Social drinking was also noted as a form of job networking. Excessive drinking was described as a way to feel less nervous in social situations. Opting to not drink in social setting was common challenge due to fear of being isolated or rejected. Social environment barriers emerged most commonly among those whose AUDIT scores indicated drinking at hazardous levels at follow-up.

*“The amount of alcohol I take is not definitive because you may be there and you just decide to go and have fun. When you are with your friends you chat about development, family and if a friend knows a job somewhere he tells you about that job opportunity*...” (31 years old, male, Mbarara, AUDIT scores: 8 to 9)

Easy access to alcohol was identified as a barrier to reducing alcohol use. Participants reported that if their friends and others were drinking and alcohol was easily available or offered at homes, pubs and social gatherings, it was difficult to say no to drinking. Alcohol accessibility as a barrier to reducing alcohol use emerged as a theme predominantly among individuals whose AUDIT scores indicated hazardous levels of drinking at follow-up.

*“I don’t even know what to say because I am really suffering, I can’t stop drinking because they brew alcohol at [my] home also.”* (35 years old, female, Eldoret, AUDIT scores: 33 to 27)

Among those who maintained or increased hazardous drinking at follow-up assessment, work dynamics emerged as a barrier to curtailing alcohol use. A commercial sex worker reported needing alcohol to have the courage to find clients. She felt incapable of stopping her drinking because of her livelihood. A long-distance truck driver perceived drinking as part of the work culture. He felt that it was essential for sleep and that there was an expectation that he drinks with co-workers after work.

“*I think the amount of alcohol [I] am taking is okay because of the work I do. I need alcohol in that I need alcohol for me to have courage and morale to be able to approach my customers. I can’t say that I can stop taking alcohol because when I stop I will be forced to stop this work [sex work]. And if I stop working, what will I eat?*” (28 years old, female, Kisumu, AUDIT scores: 18 to 37)

A number of participants whose hazardous alcohol use remained stable or increased at the follow-up assessment did not perceive their alcohol consumption as problematic and expressed a lack of motivation to stop drinking. There was a perception of feeling in control of their drinking and ability to function.

“*Yeah, I feel it is okay because even after taking alcohol, the next day I can still go about my duties normally and even that night after taking alcohol I can just do any other thing that I wanted to do.”* (29 years old, female, Kisumu, AUDIT scores: 15 to 17)

### Patient recommendations to help curtail problematic alcohol use

Participants offered advice to help others reduce problematic drinking. There was consensus that self-initiative and willpower were foremost, and making a conscious decision to stop or reduce drinking was central for those who had become non-hazardous drinkers.

*“… one has to make his/her own decision to quit taking alcohol, if I was still taking alcohol while using ARVs I could be very weak and not very healthy as I am now.” (*55 years old, female, Eldoret*,* AUDIT scores: 18 to 0)

It was suggested that HIV health care workers should offer ongoing education about alcohol use and check in about drinking concerns during follow-ups with patients. Participants also suggested that clinics would be the place to provide help to reduce or stop drinking since it is where they go for their routine care.

*“Help should be from the clinic because [it] is where I seek information.”* (32 years old, male, Kisumu, AUDIT scores: 32 to 21)

There was agreement that individual or support group counseling would be beneficial, although some concerns about trust and confidentiality were expressed within groups. Participants thought support groups would be beneficial because they advocate for healthy living and provide an avenue for sharing experiences and building social support. Support from the spiritual community and elders was also recommended for their encouraging and steadfast support. A rehabilitation center was suggested by one, but several indicated that the time away from work and cost would not make rehabilitation centers a very viable option.

“*Counseling in a support group would be best because [you] can share ideas*.” (43 years old, male, Kisumu, AUDIT scores: 22 to 11)

## Discussion

Seventy percent of PLWH participants, predominantly males, continued to drink at hazardous levels at follow up, about four years, after their initial AUDIT, pointing to the need for more attention and resources to address hazardous drinking among PLWH. Encouragingly, 30% of PLWH participants had reduced alcohol consumption to non-hazardous levels at follow up and 19% of those who continued to drink hazardously had reduced their drinking, illustrating motivation to drink less among nearly half of the participants. When exploring facilitators to address hazardous drinking, our study found that HIV diagnosis and treatment was the primary motivation to curtail alcohol use. Learning about HIV status and starting ART may act as a catalyst to personal decision-making about health and longevity; weighing the benefits of adherence to life-saving ART versus the risks of heavy drinking. Other regional studies corroborate HIV infection and treatment being pivotal to behavior change in drinking; one study found that HIV status was the primary reason for reducing alcohol consumption and another found PLWH who had been drinkers and started ART had stopped drinking within three years [14, 49].

Other common facilitators to reduce drinking were recommendations from HIV health care workers and encouragement from social support networks. Health care workers in this HIV setting were instrumental in educating patients about the harms of alcohol use on ART functionality and adherence; moreover, their recommendations to curb alcohol use were influential among those who had reduced their consumption. The information from health care workers appeared to deepen participant knowledge and helped influence behavior change. Other studies support HIV health care workers’ influential role in reducing risky behavior [50]. The ongoing support and encouragement from social support networks were also influential in reducing alcohol consumption. This is supported by a study in Uganda which found that social support may reduce hazardous alcohol use among PLWH [51, 52]. The role of social support may be of particular importance as it counteracts the social expectations and accessibility of alcohol. The desire to improve their economic situation also emerged as facilitator to curb alcohol consumption. Financial strain was a theme identified by another study in Kenya as motivation to stop drinking [53].

This study also found that the key barriers to reducing alcohol use among those who continue to drink hazardously centered on stress, social environment and accessibility of alcohol, with symptoms of alcohol dependency being an overarching barrier for those who continued drinking at hazardous levels. Drinking was deployed by participants as a coping mechanism to temporarily escape problems related to health, family, work and finances, which is corroborated by other studies, including one in Kenya [53, 54]. The pressure of social norms to fit in and drink when surrounded by friends, colleagues and family members is supported by a study in Kenya describing the negative influence of those who undermine attempts to stop drinking [53]. The accessibility of alcohol, for instance having alcohol brewed at home or working at club, is corroborated by another study which found high accessibility of alcohol as a key barrier to reducing alcohol use [54]. Among PLWH who continued drinking at hazardous levels, alcohol dependency was extremely difficult to address given the stack of other barriers and absence of appropriate tools or interventions to successfully abstain from alcohol use. Although health care workers were instrumental in educating participants about ART functionality and alcohol use, there was a distinct absence of counseling, tools or referrals shared by health care workers to help participants address problematic drinking. Interventions are needed to equip PLWH drinking hazardously with the skills and resources to combat these barriers successfully.

Other studies have identified barriers that interfere with health care workers’ ability to address alcohol use, including being overburdened and having no time to screen for alcohol, limited disclosure of alcohol consumption, perception that it is not a priority issue compared to other clinical care needs and having a specialist for problematic drinkers [55–57]. One study found that only about half of PLWH who were problem drinkers had talked to a HIV health care worker about their alcohol use in the past year [58]. Health care workers routinely monitor patient clinical care, yet frequently overlook hazardous alcohol use, which is unfortunate given its association with lapses in adherence and retention, poor health outcomes and HIV transmission [16, 17, 20, 42, 59]. Barriers to health care workers addressing alcohol use with their patients may center around deficiencies in counseling skills, tools and resources, as well as appropriate outside referral options [56, 60–62]. A study in South Africa identified provider knowledge gaps related to identifying levels of hazardous alcohol consumption, how to address or treat hazardous drinking, and clarity on their role in addressing problematic drinking [61]. Other studies found that health care workers displayed stigma, poor communication and engagement during clinical care with PLWH who drink alcohol problematically [42, 62–66]. Stigmatization by providers is pronounced and extends to community health workers who show similar attitudes towards PLWH who drink, to the extent that PLWH have expressed being too fearful to disclose their alcohol use [42, 65]. Yet, health care workers in the HIV setting are in an optimal position to address hazardous drinking; they are diagnosing patients with HIV, uncovering problematic alcohol use when exploring treatment readiness, and monitoring patient health over time. In this study, participants preferred receiving support for alcohol reduction from their routine HIV health care workers; which is corroborated by studies in Zimbabwe, South Africa and Uganda [14, 40, 57]. Another study in South Africa revealed a preference for trained peers, individuals who have similar experiences. Use of peers is a valuable approach as it allows for task-shifting away from overburdened health care workers [65]. Trained community health care workers can also be leveraged to screen for alcohol use when following up with PLWH who have missed visits or fallen out of care [65]. Studies have also demonstrated a desire for more alcohol treatment literacy for both health care workers and PLWH and shown the acceptability of alcohol reduction interventions in the HIV care setting [40, 65]. It is important to strengthen infrastructure with approaches that don’t overburden the health system and address patient needs by building the capacity of both professional and lay health workers to provide non-judgmental screening and treatment interventions for hazardous alcohol use in PLWH [50, 67].

### Strength and limitations

This study provides a deeper understanding of what facilitates PLWH with a history of hazardous drinking to reduce or stop hazardous drinking, including HIV diagnosis as a catalyst for change, the influential role health care workers play, and the service gaps that persist in addressing hazardous alcohol use among PLWH. Examining PLWH experiences based on their AUDIT score at follow-up adds strength and value by discerning differences in what facilitates and hinders behavior change in alcohol use.

The study is not without limitations. The views and experiences are from a relatively small number of PLWH in HIV care and may not be generalizable. Although experienced qualitative researchers carried out the interviews and garnered participant trust and confidentiality using validated measures, there is a potential for social desirability or recall bias with self-report.

## Conclusion

Although most PLWH participants continued to drink hazardously at follow-up, facilitators to curb hazardous alcohol use were identified among the third who had successfully reduced alcohol consumption. Their HIV condition, desire for longevity, and health care worker and social support systems were identified as key facilitators in reducing alcohol consumption. Interventions that capacity-build professional and lay health care workers with the skills and resources to better address hazardous alcohol use, along with alcohol cessation support within existing peer support group structures, should be explored.

## Data Availability

The datasets used and/or analyzed during the current study are available from the corresponding author on reasonable request.

## Abbreviations

AMPATH: Academic Model Providing Access to Healthcare
ART: Antiretroviral therapy
AUAC: Alcohol Use Assessment Cohort
AUD: Alcohol Use Disorder
AUDIT: Alcohol Use Disorder Identification Test
DALY: Disability-adjusted life year
EA-IeDEA: East Africa International epidemiology Databases to Evaluate AIDS
FACES: Family AIDS Care and Education Services
IREC: Institutional Research and Ethics Committee
ISS: Immune Suppression Syndrome clinic
KEMRI: Kenya Medical Research Institute
PLWH: People living with HIV
SERU: Scientific Ethics Review Unit
SSA: Sub-Saharan Africa
WHO: World Health Organization
